# Enhancing Electronic Availability of Hospital Records Following Interhospital Transfer From Emergency Departments to a Veterans Affairs Hospital

**DOI:** 10.1093/milmed/usaf288

**Published:** 2025-06-25

**Authors:** James O Jordano, Maureen Fausone, Michael R Cauley, Melissa Rubenstein, Terra Swanson, Kelly Sopko, Michael J Ward

**Affiliations:** Department of Emergency Medicine, Vanderbilt University Medical Center, Nashville, TN 37232, United States; Department of Medicine, University of Illinois Chicago, Chicago, IL 60607, United States; Department of Emergency Medicine, Vanderbilt University Medical Center, Nashville, TN 37232, United States; Center for Research and Innovation in Systems Safety, Vanderbilt University Medical Center, Nashville, TN 37232, United States; Department of Biomedical Informatics, Vanderbilt University Medical Center, Nashville, TN 37232, United States; Department of Emergency Medicine, Vanderbilt University Medical Center, Nashville, TN 37232, United States; Division of Pulmonary Diseases and Critical Care Medicine, University of North Carolina, Chapel Hill, NC 27599, United States; Section of Hospital Medicine, Veteran’s Affairs Tennessee Valley Healthcare System, Nashville, TN 37212, United States; Division of Internal Medicine and Public Health, Vanderbilt University Medical Center, Nashville, TN 37232, United States; Department of Emergency Medicine, Vanderbilt University Medical Center, Nashville, TN 37232, United States; Department of Biomedical Informatics, Vanderbilt University Medical Center, Nashville, TN 37232, United States; Geriatric Research, Education, and Clinical Center, Tennessee Valley Healthcare System, 1310 24th Nashville, TN 37212, United States

## Abstract

**Introduction:**

With regionalization of specialized care and consolidation of rural hospitals, safe and efficient care transitions via interhospital transfers, particularly for patients in the emergency department (ED), are of paramount importance to ensure optimal patient outcomes. Transferred patients are at higher risk for mortality, longer hospital stays, and increased costs. Complete documentation is central to high quality care transitions yet is infrequently completed. Incomplete documentation may harm patients by fragmenting care. Since the passage of federal legislation, non-Veteran Affairs (VA) emergency care has skyrocketed. However, the lack of a standardized process for medical record sharing following non-VA ED visits and subsequent interhospital transfer risks care interruption and therefore patient safety. We sought to evaluate the existing process and how standardization may affect the timeliness and availability of Veteran hospital records from non-VA hospitals.

**Materials and Methods:**

This was a quality improvement project conducted at a single, urban, community, level 1 complexity VA—the VA Tennessee Valley Healthcare System, Nashville Campus (TVHS-Na). Before this project, paper medical records related to non-VA emergency care were given to the primary team by the transferring ambulance service. Paper records were then uploaded to the document imaging system at each team’s discretion, typically after hospital discharge. To understand the problem and flow of records, we conducted a contextual inquiry. From there, we developed and implemented a standardized process to receive and upload electronic health records before the patient arrived at the hospital for all daytime weekday transfers. After-hours transfers maintained existing processes. The project had a Plan-Do-Study-Act design, informed by the VA Quality Enhancement Research Initiative Roadmap.

The project was approved as a quality improvement project by the local institutional review board. Interhospital transfers were tracked from January 2024 to September 2024. All transfers from a non-VA ED to TVHS-Na as a direct hospital floor admission were included. The primary outcomes were time until availability of scanned records and percent of transfers with uploaded records as identified in the document imaging system.

**Results:**

Through a contextual inquiry with stakeholders, we identified that intervention at the time of transfer acceptance was possible with the help of transfer coordinators. As part of the novel intervention, coordinators would ask transferring hospitals for electronic transmission of certain optional documents. This would occur before the physical transfer of the patient. Over the project’s course, there were 157 interhospital transfers from non-VA EDs that met eligibility. Before implementation, the median (interquartile range, IQR) time to document availability was 33 (24, 36) days. The proportion of transfers with uploaded records at this time was 40% (13/32). Ten transfers occurred during the “washout phase” during which the intervention was implemented. After implementation, the median (IQR) time until upload improved to 0 (0, 0) days. The proportion of transfers with uploaded records also improved to 51% (59/115).

**Conclusions:**

Implementing a simple, standardized process increased the number of transfers with available records and reduced the time until the electronic availability of those records. However, after-hours transfers remain a target for future intervention.

## INTRODUCTION

With the regionalization of specialized care^1^ and the consolidation of rural hospitals,^2^ interhospital transfers are a key source of hospital admissions that need timely and safe access to care. However, transferred patients are at risk for higher mortality, longer hospital stays, and higher costs.^3^ With the majority of clinical data lacking interoperability and being siloed in separate electronic health records, seamless transfer of records is crucial to minimize potential risks incurred during interhospital transfer.^4^ However, hospital transfer processes vary widely, lack standardization or clear guidelines, and may contribute to incomplete transfer documentation. For example, in 1 sample of 32 tertiary care facilities with an estimated 700 transfers per month, only 29% had documentation available before transport.^5^ Interhospital records, commonly in the form of paper records, are often the only data available during the transition of care. Even when obtained, such records typically do not make it to the clinicians caring for the patient.^6^ Patients who arrive without such records force the receiving hospital to recreate the clinical encounter and possibly delay care.^5^

The Veterans Health Administration is particularly susceptible to a lack of medical record sharing between institutions. A major component of this burden is records from non-Veteran Affairs (VA) interhospital transfers—where a Veteran presents to a non-VA emergency department (ED) and is transferred to the VA. With approximately $6 billion spent annually on non-VA care, interhospital transfers continue to be an important source of access to care for Veterans.^7^ Even when records are obtained by the VA, barriers remain. In 2019, the VA Office of Inspector General found there were significant medical documentation backlogs. At that time, at least “5 miles” of records for nearly 600,000 Veterans were not scanned into VA record systems.^8^

The lack of a standardized record upload process may be contributing to this backlog. The VA Tennessee Valley Healthcare System, Nashville Campus (TVHS-Na) lacked a standardized process for uploading the patient’s associated transfer documentation. We sought to determine if creating a uniform process during interhospital transfers would improve the timeliness and availability of non-VA hospital records for transferred Veterans.

## MATERIALS AND METHODS

### Study Subjects, Design, and Setting

We conducted a prospective single-institution quality improvement project using a Plan-Do-Study-Act design, informed by the VA Quality Enhancement Research Initiative Roadmap.^9^ The study was conducted at TVHS-Na, an urban, community, level 1 complexity VA in Nashville, TN, United States. On average, it receives approximately 5 medical transfers per week, directly admitted to hospital floors. Inpatient floor teams consist of an attending physician and resident physicians, all of whom rotate biweekly. Resident physicians are responsible for the receipt and upload of outside paper records. Anecdotally, transferred medical records were not consistently available in a timely manner, and provided motivation for the existing project.

### Data Collection

**Figure 1.**
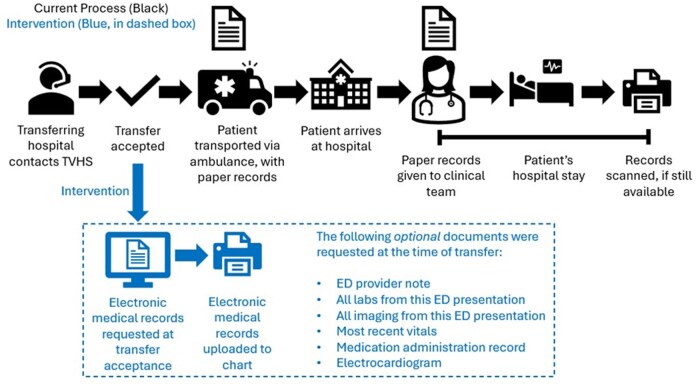
Pre-intervention flow (in black, outside of dashed box) and post-intervention flow (in blue, in dashed boxed) of medical records for daytime non-veteran affairs emergency department transfers, with the list of optional requested documents.

A multidisciplinary team consisting of attending physicians, fellows, residents, nurses, transfer coordinators, and administrative officers participated in a contextual inquiry to understand the flow of records. We delineated each step of the transfer process, discussed possible points at which we could intervene, polled possible barriers to intervention, and noted additional roles that should be included in the discussion. We additionally enumerated the different ways records could be uploaded, who is approved to upload records, and steps commonly associated with delays in record upload. Next, we designed and implemented the intervention, in which transfer coordinators request electronic records before patient transport. Details of the pre-intervention and post-intervention processes are discussed below and in **Figure 1**.

To monitor the intervention, we tracked eligible interhospital transfers originating from a non-VA ED as a TVHS-Na hospital floor admission. These transfers were already being tracked as part of a separate VA-funded study. Patients transferred from a non-VA hospital floor, assisted living facility, another VA, outpatient clinic, or to the intensive care unit were excluded. These transferring facilities have different records available at the time of transfer as well as different record transfer capabilities. Thus, we focused on ED transfers to improve feasibility and evaluation of the intervention. A record of all outside hospital transfers from January 2024 to September 2024 was evaluated. Transfers from January 2024 to February 2024 were categorized as pre-intervention. The intervention began on March 1, 2024. March 2024 served as a “washout phase” to optimize the intervention. April 2024 through September 2024 were categorized as post-intervention. A postdoctoral research fellow completed a non-medical chart review of each transfer. Collected data included transfer date and the date records were scanned into the document imaging system. Records were reviewed regularly to capture record uploads, with the maximum review at least 30 days post-transfer date.

### Analysis

Observational data collected from the contextual inquiry with stakeholders were reviewed to identify major themes. The research team discussed the themes to inform intervention design. After discussion, the team reengaged stakeholders for further feedback. The team repeated this process until all themes were addressed by the intervention. The primary quantitative outcome was the time until availability of scanned records. Secondarily, we assessed the proportion of transfers that had outside records uploaded within 30 days of transfer. The analysis was primarily descriptive. The project was approved by the local institutional review board as a quality improvement project.

## RESULTS

### Existing Transfer Process

The pre-intervention interhospital transfer process is described in **Figure 1**. We found that the daytime transfer process was different than after-hours. Transfer coordinator nurses oversee daytime transfers, while nonclinical administrators oversee nighttime transfers; the 2 groups have different permissions and capabilities in their respective transfer processes. With different transfer processes for daytime and after-hours transfers, we focused on daytime transfers to improve the feasibility of the project.

In the pre-intervention process, after the transfer was accepted by the physician, transfer coordinators facilitated patient transport to TVHS-Na. Patients were transported with paper medical records from the non-VA ED. The transferring ambulance service providers gave the records to the inpatient team upon arrival. Documents were retained and uploaded to the document imaging system at each team’s discretion. This was most often after patient discharge. During the hospital stay, records were at risk of becoming disorganized, lost, or shredded before upload. This process could potentially delay document uploads for weeks after patient arrival if ever done. We also identified that transfer coordinators had the ability to review and upload records. Furthermore, we mapped an upload channel that would support urgent upload (within 48 hours) rather than routine upload (within 30 days), while ensuring record confidentiality would be maintained throughout the process.

### Intervention

After completion of the contextual inquiry, we designed the intervention that is represented in **Figure 1**. The key barrier, and the target of the intervention, was the time to upload medical records during the transfer process. Therefore, the new intervention process had the transfer coordinators request outside electronic medical records at the time of transfer acceptance, before patient arrival. A list of relevant medical records was compiled (**Figure 1**, blue dashed box). To prevent a delay in patient transport that might hinder patient care, the medical record request was optional. Once received, the transfer coordinator scanned the records to upload them to the document imaging system. Post-implementation, medical records that were not sent per the new intervention still could be manually uploaded via paper record receipt as they were pre-intervention. Therefore, the intervention expanded the responsibility of record receipt/upload beyond residents to also include full-time transfer coordinators.

**Figure 2.**
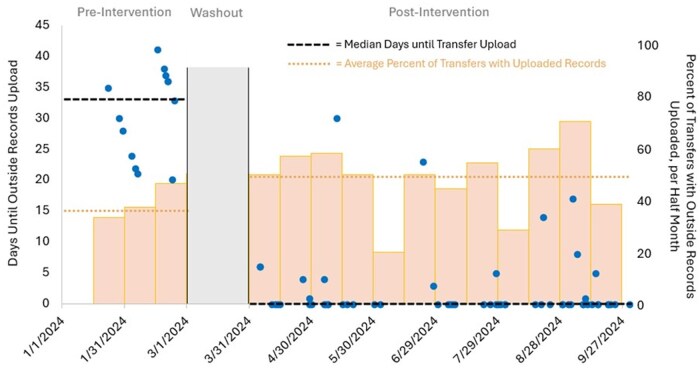
Days until upload of outside hospital records (blue dots) and percent of transfers with electronic records available (bars). Median “Days Until Outside Records Upload” are denoted pre-intervention and post-intervention (black dashed lines). Average percent of transfers with records uploaded within 30 days (orange dotted lines) are also noted. The washout period is marked by the gray bar. Pre-intervention median to until upload (interquartile range [IQR]) is 33 (24, 36) with an average percent upload of 40%. Post-intervention median (IQR) is 0 (0, 0) with an average percent upload of 51%.

Between January 16, 2024 and September 30, 2024, there were 157 interhospital transfers from non-VA EDs that met eligibility. There were 32 transfers before intervention implementation, 10 during the washout period in March 2024, and 115 transfers post-intervention. Pre-implementation, the median (interquartile range, IQR) time to document availability was 33 (24, 36) days (see **Figure 2**). The proportion of transfers with uploaded records pre-implementation was 40% (13/32). Post-implementation, there were 115 transfers. The median (IQR) time until upload improved to 0 (0, 0) days, indicating same-day upload (**Figure 2**). The proportion of transfers with uploaded records also improved to 51% (59/115). Most of the uploaded records, 76% (44/59), were uploaded within 24 hours of arrival.

## DISCUSSION

Electronic availability of outside transfer documents was frequently delayed beyond a clinically relevant period, if the documents were ever uploaded. We identified that the lack of a standardized process contributed to this problem. After a contextual inquiry with stakeholders, we implemented a simple, standardized process at the point of transfer acceptance that increased the number of transfers with available records and reduced the duration until the electronic availability of the records. Lack of communication between hospitals via transferred medical records represents a potential barrier to safe and high-quality care transitions. A prior study assessing handoff practices among 32 tertiary care centers reported only 29% of transfers had referring center clinical documentation available before transport,^5^ underscoring the generalizability of this problem. This study recommended several possible innovative practices to improve interhospital transfers, with 1 being a “health and information management department responsible for obtaining and scanning outside records into (electronic medical record).”^5^ We found substantial improvement with a similar intervention. A prior study found that higher transfer documentation completeness was associated with reduced in-hospital mortality, reduced adverse events, and reduced duplication of labor.^10^ Moreover, this approach also makes the transferring hospital medical records available to all providers, not just those immediately caring for the patient.

Despite the improved availability of transfers with electronic documentation, one-in-two transfers remained without uploaded records. This likely occurred for multiple reasons. First, we did not mandate electronic transmission of records before transfer. We were concerned acceptance delays secondary to lack of transfer records would affect the clinical care of the patient. Second, we only implemented our protocol for daytime transfers via the transfer coordinators. Administrative officers coordinate after-hours transfers through a different process. Transfer coordinators, being clinical staff, have permission to review transferred records and to scan uploads. Non-clinical administrators do not have these permissions. They also are tasked with other responsibilities during their shifts not shared by transfer coordinators. A modified intervention will be necessary to cover after-hours transfers after considering the limited clinical permissions and broad scope of responsibilities of administrative officers. However, implementation during the daytime may facilitate use of documentation upload after hours. Third, extra-institutional barriers to timely upload remain. These include the varying ability of transferring facilities to collect/transmit records and patient acuity affecting time to gather necessary records. Further efforts in interhospital communication and electronic health record documentation may facilitate systemwide improvement, from which ­interhospital documentation upload practices may also benefit.

This intervention had multiple positive attributes that may help long-term sustainability. The intervention shifted responsibility over outside records from residents who rotate biweekly to transfer coordinators who are present consistently and are employed by the VA. Also, electronically capturing the records, this diminishes reliance on paper records that can be completely or partially lost or become out of order. Furthermore, the intervention was integrated into the existing workflow, adding minimal additional time and steps to the established process.

Despite the strengths, there were a few notable limitations worth considering. First, this project was conducted at a single VA tertiary care center in the Southeast, and the findings may not be generalizable to other locations. Second, we only evaluated patients transferred from outside hospital EDs sent as direct general medicine floor admissions; we did not include patients admitted from inpatient services, nursing facilities, or outpatient clinics as these represent different patient populations, record availabilities, and transfer processes. However, these represent future directions for evaluation.

## CONCLUSION

The implementation of a standardized documentation process for interhospital transfers as a quality improvement project substantially reduced the duration until electronic availability for daytime interhospital transfers while increasing the proportion of these transfers with electronic records available. Future investigation is needed to examine the generalizability of these findings, the after-hours processes, and the impact on patient outcomes.

## Data Availability

The data that support the findings of this study are not openly available because of reasons of sensitivity and are available from the corresponding author upon reasonable request.

## INSTITUTIONAL CLEARANCE

Institutional approval is granted by the local institutional review board that oversees the Tennessee Valley Healthcare System Veteran’s Affairs.
